# Interaction of Memantine with Homocysteine on the Apoptosis in the Rat Hippocampus cells

**Published:** 2012

**Authors:** Amin Ataie, Ramin Ataee, Mohammad Shadifar, Sima Shahabi, Seyed Mohsen Aghajanpour, Younes Hosseinpour

**Affiliations:** 1 *Cellular and Molecular Biology Research Center, Babol University of Medical Science, Babol, Iran.*; 2 *Pharmaceutical Sciences Research Center, Department of Pharmacology and Toxicology Mazandaran University of Medical Sciences, Sari, Iran.*; 3 *Pastor Institute of Iran, Tehran, Iran.*

**Keywords:** Homocysteine, apoptosis, memantine, alzheimer’s disease, hippocampus, NMDA receptor, oxidative stress

## Abstract

It has been hypothesized that elevated plasma Homocysteine (Hcy) plays a role in the pathogenesis of Alzheimer’s disease (AD) and age-related cognitive decline. The mechanism of Hcy neurotoxicity in the brain is controversial as well Hcy is a ligand of NMDA receptor. Memantine, an uncompetitive antagonist of N-methyl-D-aspartate (NMDA) receptors approved for the treatment of moderate to severe Alzheimer's disease.

Hcy was injected 0.5 μmol/μl in the hippocampus of the rat brain and Memantine hydrochloride was injected 10mg/kg intraperitoneally 1 hour prior to Hcy injection. After five days, rats were killed and whole brain were taken out, fixed, and embedded in paraffin. The slices of the rat brain were prepared and immunohistochemical analysis was done to reveal the protein expression of Bax, Bcl-2, and the activation of Caspase 3 in the rat hippocampus layers. Results showed significant increase of Bax and Caspase-3 immunoreactivity in hippocampus of rat brain in Hcy group. Also an increase in Bax/Bcl-2 ratio in rat hippocampus cells .Memantine pretreatment could not change the levels of Bax, Bcl-2, Caspase-3 significantly in rat’s hippocampus cells.

These findings suggest that Memantine could not antagonize Hcy – induced Apoptosis. Hcy may induce apoptosis via the other oxidative stress mechanism in the rat brain. potential. It may therefore be interesting that he barberry fruit extracts has the unique capacity to quench free radicals.

The Etiology of Alzheimer’s Disease are related to some major risk factors including Apolipoprotein E_4_, A_2 _Macroglobolin, Persenilin 1,2.Amyloid Precursor Protein (APP) and many neurotoxins that accelerate Beta-Amyloid deposits in the brain and impaired memory process.

Increased concentration of Homocysteine in human blood more than 14 μM is called *Hyperhomocysteinemia* and has been known as a risk factor in cardiovascular and neurodegenerative disease (1).

Hyperhomocysteinemia is a risk factor for stroke, Alzheimer’s and Parkinson’s diseases, schizophrenia, depression, renal diseases, leukemia and diabetes (2, 3). Homocysteine (Hcy) is a sulfur-containing amino acid derived from methionine. Moderate to severe hyperhomocysteinemia can be caused by deficiencies of folate or vitamin B12, genetic defects in the metabolism of methionine, certain drugs and renal disease (4).

Elevated blood levels of Hcy can potentially inhibit methyl transferase reactions involving DNA, proteins, phospholipids and catecholamine neurotransmitters (5). Substantial data from various epidemiological studies found elevated blood level of Hcy in patients with mild cognitive impairment, Alzheimer’s disease, depression and schizophrenia suggesting the association between hyper-homocysteinemia and those neuropsychiatric diseases (5).

However, the mechanism by which elevated blood concentrations of Hcy are related to the pathogenesis of cognitive dysfunction remains incompletely understood. The thiol group of Hcy is readily oxidized in plasma and culture medium, resulting in the generation of reactive oxygen species (ROS). Moreover, Hcy has the ability to inhibit the expression of antioxidant enzymes such as glutathione peroxidase (GSH-Px), and super oxide dismutase (SOD) (6). Hcy is an excitatory amino acid, which markedly enhances the vulnerability of neuronal cells to excitotoxicity and oxidative injury (6). Hcy is a glutamate agonist, which causes an increase in Ca2+ influx via the activation of the NMDA class of excitatory amino acid receptors, which in turn, results in neuronal cell death and apoptosis (7).

Memantine, a drug used clinically for the treatment of moderate to severe Alzheimer’s disease is a moderate-affinity, uncompetitive antagonist of NMDA receptors (8, 9). It has been shown that Memantine-treated rats are less neurodegeneration induced by Ab1–40 injections into the hippocampus (10).

The purpose of this study was to test the hypothesis that Memantine treatment prevents apoptosis of hippocampal neurons in rats that were intra hippocampal injected with Hcy. We further hypothesized that Hcy injection would induce detectable changes in intracellular levels of one or more Caspase or Bcl-2-related proteins (Bcl-2, Bax) and that such changes would be attenuated by Memantine treatment.

## Materials and Methods

***Drugs and Biochemical reagents***

D-L-Homocysteine and Memantine Hydrochloride were purchased from Sigma-Aldrich, Germany. Ketamine and xylazine were obtained from ALFASAN Co., Netherlands. Hcy powder was dissolved in hydrochloric acid (1 M) and diluted with PBS (Sigma-Aldrich). The pH of the solution was regulated at 7.4 by the addition of 0.1 N NaOH. Solutions of Hcy were prepared freshly at a concentration of 0.5 μmol. Memantine hydrochloride was dissolved in distilled water at 10 mg/ml concentration.

**Animals**

Adult male Wistar rats were taken from animal house of Babol University of Medical Sciences, Iran weighing between 200 and 250 g. The animals were housed at 22°C in a controlled environment with a 12:12- h light/dark cycle and were given access to standard laboratory food and water. All experiments were carried out in accordance with the National Institutes of Health guidelines 13 and were approved by the Research and Ethics Committee of Babol University of Medical Sciences We used animals groups with six animals per group. Animals of the control group received vehicle of Hcy. In the Hcy group, 1 μl Hcy (0.5 μmol/μl) was injected intra hippocampus, and saline was injected (i.p.) one hour prior to Hcy injection. In the Hcy-Memantine group, Memantine HCl (10mg/kg) was injected (i.p.) prior to Hcy injection.

**Intra hippocampus injection**

The rats were anaesthetized with Ketamin- Xylosin (10 mg/kg i.p.) and placed in a stereotaxic frame. The rat skull was orientated according to Paxinos and Watson stereotaxic atlas (11). After a sagittal incision, the bregma suture was located and holes were drilled with an electrical drill at the following co-ordinates; 3.3 mm posterior to bregma, 2.6 mm lateral to the sagittal suture and 3.6 mm ventral. Care was taken not to damage the meninges. A Hamilton syringe with a cannula of diameter of 0.3 mm was used to inject 1 μl of solutions of Hcy 0.5 M or its vehicle (PBS). The injection was carried out in the left and right dorsal hippocampus at a rate of 1 μl per 2 min. The cannula was left in situ for a further 5 min following Hcy injection to allow passive diffusion from the cannula tip and to minimize spread into the injection tract. The cannula was then slowly removed and the scalp was then closed with sutures. Animals were kept warm until recovery from the anesthesia.

**Hippocampus Histopathologic analysis**

Removed hippocampal tissues were fixed in 10% neutral buffered formaldehyde for 24h, embedded in paraffin and cut into at 3–4 μm thick sections by a microtome (Leica SM2000R, Germany). The tissue sections were deparaffinized in xylene. The slides were used for Immunohistochemistry process.

**Immunohistochemistry**

Immunohistochemistry (IHC) for Bax, Bcl-2, and cleaved Caspase 3 were carried out on formalin-fixed, paraffin-embedded sections and according to the manufacturer’s instructions provided for each antibody. Sections were deparaffinized and rehydrated. Antigen retrieval was carried out by microwaving in citrate buffer (pH 6) for 1 and 2×5 min for Bax and Bcl-2, respectively. Cleaved Caspase-3 antigen unmasking was performed by microwaving in EDTA/Tris buffered saline Tween-20 (TBST) for 10 min. The sections were quenched with 3% hydrogen peroxide (H_2_O_2_) in absolute methanol and blocked with 10% normal goat serum (NGS) + 1% bovine serum albumin in phosphate-buffered saline (PBS) for Bax and Bcl-2 and with 5% NGS+ in TBST for cleaved Caspase- 3. Primary antibodies were applied overnight at 4°C. These were either: Bax rabbit polyclonal antibody (abcam, 1/100), Bcl-2 rabbit polyclonal antibody (abcam, 1/100), and cleaved Caspase- 3 rabbit monoclonal antibody (Cell Signaling, 1:100). 

The sections were washed and then incubated with a ready-to-use anti rabbit secondary antibody from Dako (EnVision Plus), and color reaction was developed using diaminobenzidine as the chromogen. The slides were then counterstained with hematoxylin, dehydrated using graded alcohols and xylene, and mounted with Permount mounting medium (Entellan, MERK).

**Statistics**

The Bax, Bcl-2, Caspase3 immunostaining, were determined at each control, homocysteine, and Memantine-homocysteine groups by using the mean scales of Bax and Bcl-2 in the same animal. All of the data are presented as means ± standard error of the mean (SEM) and analyzed by t tests or one-way analysis of variance as appropriate using Graph Pad Prism Software (version 5). p<0.05 was considered statistically significant.

## Results

**Effects of Homocysteine on Bax and Bcl-2 Protein Levels**

**Fig 1:**
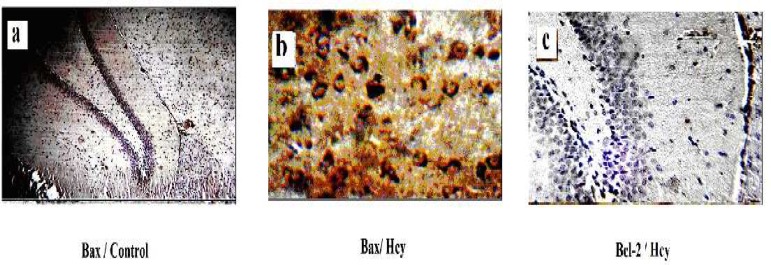
Effect of Hcy (0.5µmol) on Bax and Bcl-2 protein expression in rat hippocampus with IHC method a-control group, b-Hcy group, c-Hcy group

To determine whether Hcy leads to changes in Bcl-2 family protein levels, we examined the Bcl-2 and Bax protein immunostaining. As shown in Figure 1a, Bax protein was minimally detected in different hippocampus layers in control animals. However, in Hcy-treated rats, Bax immunostaining significantly increased in hippocampus layers (Fig. 1b) (F2, 15=23.89, P<0.001).

**Fig 2:**
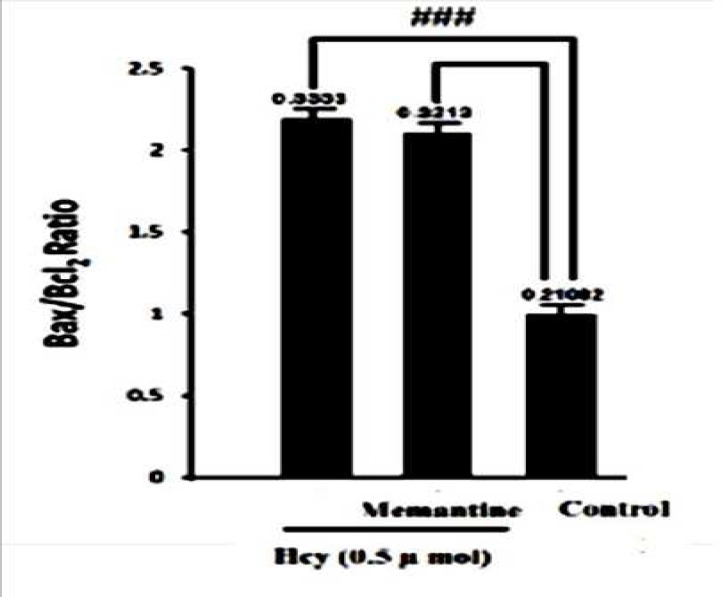
Interaction of Memantine Hcl (10mg/kg) with Hcy (0.5µmol) on Bax/Bcl-2 ratio in rat hippocampus. Data are mean values (n = 8). ### P<.001 for difference from control group

**Fig 3:**
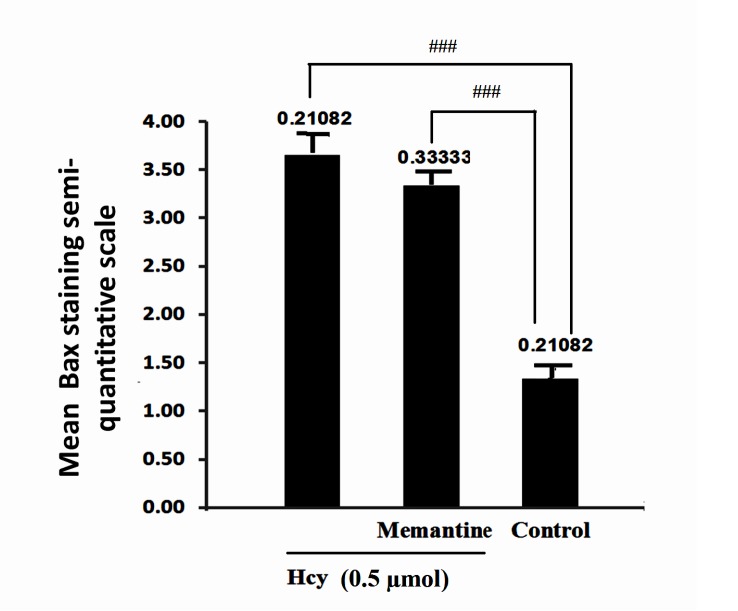
Interaction of Memantine Hcl (10mg/kg) with Hcy (0.5µmol) on Bax expression in rat hippocampus. Data are mean values (n = 8). ### P<.001 for difference from control

**Fig 4:**
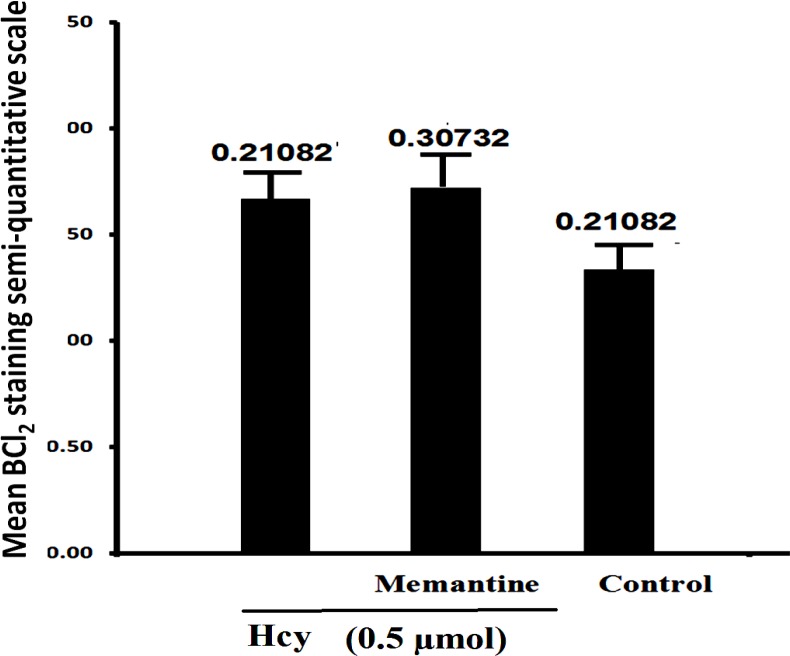
**. **Interaction of Memantine Hcl (10mg/kg) with Hcy (0.5µmol) on Bcl-2 expression in rat hippocampus. Data are mean values (n = 8).

In contrast with Bax, Bcl-2 staining showed a vigorous expression of this anti apoptotic protein in control group, and this refers to the constitutive expression of Bcl-2 protein in normal conditions, as shown in Figure 1c. Hcy exposure slightly increased Bcl-2 immunostaining. The Bax to Bcl- 2 ratio was calculated for hippocampus tissue as explained above. As shown in Figure. 2, Hcy treatment significantly increased this ratio (F2, 15=34.169, P<0.001). In addition, there was no significant change in Bax/Bcl-2 ratio with Memantine pre-treatment in Hcy groups (Fig. 2, 3, 4).

**Effects of Homocysteine on Activated Caspase 3 Immunostaining**

**Fig 5:**
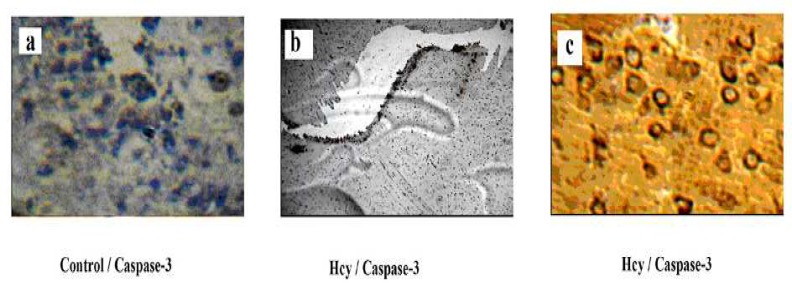
Effect of Hcy (0.5µmol) on Caspase-3 protein expression in rat hippocampus with IHC method a-control group, b-Hcy group, c-Hcy group.

**Fig 6:**
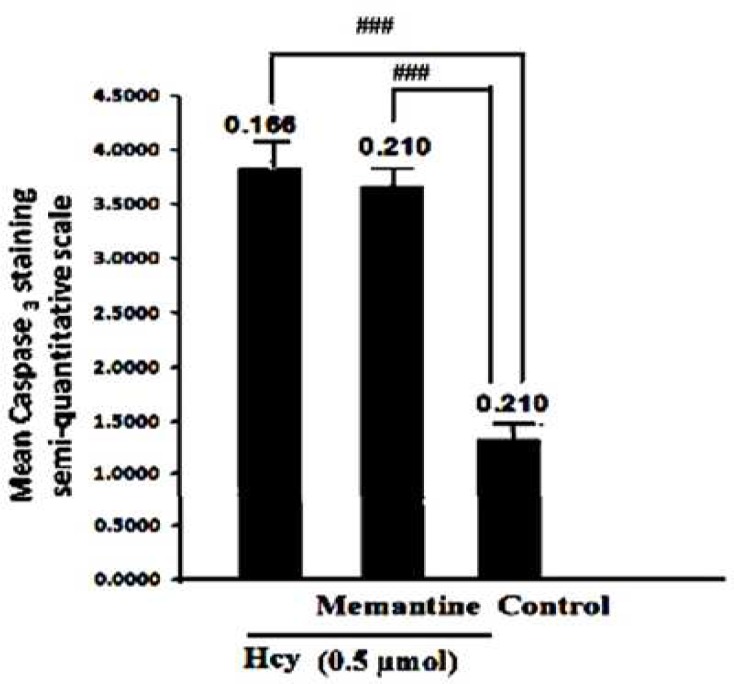
Interaction of Memantine Hcl (10mg/kg) with Hcy (0.5µmol) on Caspase -3 expression in rat hippocampus. Data are mean values (n = 8).

To confirm whether the increase of Bax/Bcl-2 ratio does lead to the activation of apoptotic cascade, we analyzed the immunostaining of cleaved Caspase 3 in all of the experimental groups. Rabbit monoclonal antibody used in this study detects cleaved Caspase 3, the large active fragment that is derived from inactive full-length Caspase 3. As shown in Figure 5a, the degree of cleaved Caspase 3 immunostaining was low in control groups. But as expected according to the raised Bax/Bcl-2 ratio in Hcy-treated groups, active Caspase-3 immunoreactivity significantly increased as a result of Hcy toxicity (F2, 15=50.24, P<0.001)(Fig. 5b, c). Memantine pretreatment did not decrease Caspase 3 immunoreactivity significantly (Fig. 6).

## Discussion

The aim of this study was to investigate the neurotoxicity of Hcy on hippocampus cells. Hcy (0.5 µmol) was directly injected in rat hippocampus and programmed cell death (apoptosis) was analyzed by Immunohistochemistry method.

Three markers: Bax, Bcl-2 and Caspase 3 were detected and Bax/Bcl-2 ratio was also measured. For investigation the mechanism of Hcy neurotoxicity, a NMDA receptor antagonists (Memantine hydrochloride) was used prior to Hcy treatment.

The results showed that the expression of apoptosis regulatory proteins, Bax and Bcl-2, would be altered by Hcy (Figs. 1, 2). Memantine pretreatment did not change significantly apoptotic biomarkers compared with Hcy group. Furthermore it did not decrease Bax/Bcl2 ratio in hippocampus cells significantly (Fig. 2, 3, 6). It seems that Hcy express neurotoxic effect without activating NMDA receptors.

We investigated neurotoxicity of Hcy in the previous studies (12, 13). It was suggested that Hcy might generate reactive oxygen species (ROS), which attack the poly unsaturated fatty acids (PUFA) of neuronal cell membranes and induce lipid peroxidation in the hippocampus (14). Hcy may also modulate intracellular signaling, ultimately leading to neuronal death via apoptosis or necrosis (14). ROS are highly reactive with all biological macromolecules (e.g. proteins, DNA and lipids). PUFAs are extremely sensitive to free radicals due to their double bonds. ROS attack PUFAs, which initiates a chain reaction called "lipid peroxidation" (15).

In the course of this process, many detrimental metabolites, such as peroxides, aldehydes, ketones and alcoholes are generated. Peroxides and aldehydes, especially 4-hydroxy-nonenal (HNE) and MDA, are the key-factors for the initiation and the propagation of atherosclerosis, inflammation, cancer and neurodegenerative diseases (16).

The development of neuroprotective agents for the prevention of neuronal loss in acute conditions such as stroke and epilepsy or chronic neurodegenerative disorders including Parkinson's disease, Alzheimer's disease, Huntington's chorea, and motor neuron disease is currently focusing on drugs that inhibit excitatory amino acid neurotransmission or exhibit antioxidant properties (17). Low affinity NMDA receptor antagonists like Amantadine and its dimethyl derivative, Memantine, have been administered clinically for the management of Parkinson's disease, dementia, neuroleptic drug-induced side effects, and spasticity (17). These agents have only rarely induced significant psychotomimetic side effects. The NMDA receptors play a critical role in excitotoxicity and are classified as ionotropic glutamate receptors. The NMDA receptor channel is a complex molecular entity containing number of distinct recognition sites for endogenous and exogenous ligands (18). These receptors are ligand-gated, voltage-dependent ion channels, which at resting potential are blocked by a magnesium ion that is only displaced following depolarization of the neuron by the binding of glutamate to the receptor along with a co-agonist, glycine, to a modulatory site (19). Beside allowing intracellular influx of Ca_2+_, the NMDA receptor also permits exchange of Na_+ _and K_+ _across the cell membrane. However, the Ca_2+_ permeability of NMDA receptor is important to the initiation of long term potentiation as the increase of Ca_2+_ concentration activates a number of cellular and second messenger systems (20). When injected into rat hippocampus in amounts not immediately toxic, NMDA induces a gradual neurodegeneration over several hours, which can be inhibited by delayed administration of NMDA antagonists (20).

Alzheimer’s disease (AD) is an irreversible, progressive neurodegenerative disorder that occurs gradually and results in memory loss, unusual behavior, personality changes, and a decline in thinking abilities. AD is a dementia characterized by neurodegeneration; in particular, a loss of acetylcholine in the entorhinal cortex, hippocampus, ventral striatum, basal forebrain, and cerebral cortex and behavioral pathologies, such as wandering behavior (21, 22). It is now recognized that subjects with cardiovascular risk factors and a history of stroke have an increased risk of both vascular dementia and AD (22).

Elevated Hcy levels in the developing nervous system and adverse effects of folate/vitamin B12 deficiency, taken together with the fact that hyperhomocysteinemia, has prompted examination of potential roles for Hcy and deficiencies in one-carbon metabolism in age-related neurodegenerative disorders such as AD and PD (15). Hyperhomocysteinemia has been related to cerebral microangiopathy (23), endothelial dysfunction, impaired nitric oxide activity and increased oxidative stress and all factors associated with aging of the brain (23). Hcy also promotes copper-mediated and β-amyloid-peptide-mediated toxic effects in neuronal cell cultures (15) and induces apoptosis in hippocampal neurons in rats (15). Hcy can damage and kill neurons in cell culture and can increase their vulnerability to being killed by various excitotoxicity, oxidative and metabolic insults (15).

Our results suggested that Hcy may induce apoptosis and cell death in rat hippocampus. Hcy my induce oxidative stress and produce ROS that attack all biological macromolecules (e.g. proteins, DNA and lipids). It is suggested that Hcy may be a risk factor for AD and PD. Also Memantine could not antagonize Hcy neurotoxicity and not improve hippocampus cell death in our experiment.
